# Hemophagocytic Lymphohistiocytosis Associated to *Klebsiella pneumoniae* Infection: A Case Report

**DOI:** 10.3389/fimmu.2021.684805

**Published:** 2021-07-12

**Authors:** Zhiyu Zhang, Junqian Liu, Jingyue Wang, Yushi Wang

**Affiliations:** Department of Cardiology, The First Hospital of Jilin University, Changchun, China

**Keywords:** hemophagocytic lymphohistiocytosis, hemophagocytic syndrome, *Klebsiella pneumoniae*, hyperferritinemia, ferritin, intensive care unit

## Abstract

This is a case analysis of a 73-year-old Chinese man admitted to the cardiac intensive care unit (ICU) with fever and general pain. Based on the patient’s initial condition of multi-organ function impairment and increased serum ferritin, and after a series of examinations, the patient was diagnosed with Klebsiella pneumonia-induced hemophagocytic lymphohistiocytosis (HLH). Meropenem and dexamethasone were used in combination to treat the patient, and the results were very successful. In this case report, it is further suggested that *Klebsiella pneumoniae* is a possible trigger of HLH, and a combination of antibiotics and corticosteroids can be effective in treating HLH. It is also recommended that doctors in the ICU of each department should pay attention to the role of hyperferritinemia in the diagnosis of HLH, and ICU admission teams should include ferritin in their monitoring.

## Introduction

Hemophagocytic lymphohistiocytosis (HLH) is a serious, uncontrolled, and self-sustaining inflammatory syndrome (1), with two forms, primary and secondary. HLH induced by Epstein–Barr virus (EBV) infection is the most common type in children, while, HLH is commonly caused by lymphoma in adults (2, 3). It is well-known that adults with secondary HLH have extremely high mortality and a poor prognosis. Quick identification, diagnosis, and treatment of HLH are the key to reversing a tragic prognosis. This case analysis involves an HLH patient admitted to a cardiac intensive care unit (Cardiac ICU) and describes his particular clinical manifestations and onset causes. Some thoughts and experiences connected with his diagnosis and treatment are shared.

## Case Presentation

A 73-year-old Asian man was hospitalized in the Cardiac ICU with fever, thrombocytopenia, and pain in many parts of his body, including the chest, abdomen, lower back, and legs. His past medical history included hypertension and coronary heart disease, and he had three stents implanted. Home medications consisted of enalapril, aspirin, Plavix, atorvastatin, and isosorbide mononitrate sustained-release tablets. Physical examination revealed a blood pressure reading of 90/60 mmHg, a heart rate of 110 beats per minute, a body temperature of 38.9°C, normal lung sounds, and no swelling of the liver or spleen. The initial laboratory data are shown in **Table 1**. The results of computed tomography pulmonary angiography (CTPA) revealed that the enhanced lung texture in both lungs and scattered strips of high-density shadows, so a diagnosis of scattered pneumonia in both lungs was considered. Aortic computed tomography angiography (CTA) revealed atherosclerosis of the thoracic aorta and some branches. The electrocardiogram showed sinus tachycardia. Heart color Doppler ultrasound suggested that the right ventricle was enlarged and the left ventricular diastolic function was weakened. Arteriovenous ultrasound of the lower extremities revealed atherosclerosis in both limbs and an old thrombosis in the right calf muscles. Digital radiography of the knee joints revealed bone hyperplasia bilaterally. Abdominal computed tomography (CT) showed that the liver shadow was enlarged, and the abdominal fat space was cloudy. Color Doppler ultrasound of the abdomen indicated an enlarged gallbladder and cholestasis.

**Table 1:** The changes in significant laboratory test results during the whole treatment process.

Parameter	Values		Unit
	DAY1	References value	
WBC	9.67	4-10	10³/uL
Neutrophil percentage	0.96	0.4-0.75	%
Lymphocyte percentage	0.03	0.2-0.5	%
Percentage of monocytes	0.01	0.03-0.1	%
Hemoglobin	137	130-175	g/L
Thrombocytes	29	125-350	10³/uL
AST	69.2	15-40	U/L
ALT	42.4	9-50	U/L
Total bilirubin	43.9	0-26	umol/L
Direct bilirubin	34.3	0-6.8	umol/L
Lactic acid	2.3	0.5-2.2	mmol/L
Fibrinogen	4.86	1.8-4.0	g/L
Total proteins	52.1	68-85	g/L
glucose	3.67	3.9-6.1	ummol/L
BUN	16.75	3.6-9.5	mol/L
Creatinine	157.6	57-111	ummol/L
Triglycerides	2.92	0.28-1.8	umol/L
D2 polymers	53.9	0-1.0	ug/ml
CKMB	0.3n	0-0.38	g/ml
Troponin I	0.012	0.034	ng/ml
PT	12.5	9-13	s
INR	1.04	0.8-1.2	
APTT	37.0	21-33	s
PCT	14.62	0-0.5	ng/mL
CRP	332.41	0-3.5	mg/L

WBC, white blood cell; AST, aspartate transaminase; ALT, alkaline phosphatase; BUN, Blood Urea Nitrogen; CKMB, creatine kinase MB; PT, prothrombin time; INR, International Normalized Ratio; APTT, activated partial thromboplastin time; PCT, procalcitonin; CRP, C-reactive protein.

The patient was admitted to the hospital with a diagnosis of toxemia. Then, the patient received cefminox sodium for two days. A series of infectious disease examinations, including blood culture, urine culture, hepatitis B virus polymerase chain reaction (PCR), and a (1,3)-β-D-glucan test, a galactomannan antigen test, and an epidemic hemorrhagic fever virus antibody test, were all negative, except the blood culture. *Klebsiella pneumoniae* was found in the blood culture. We then switched to treatment with meropenem on the third day. Due to the patient’s further decline in platelets, a worsening creatinine level, and liver function damage, we conducted a series of tests concerning heparin-induced thrombocytopenia-related antibodies, anti-nuclear antibodies, three types of rheumatism, and anticardiolipin. The test results were all negative. A further ferritin assessment revealed a level of 2,925.3 μg/L (normal range: 20–300 μg/L), while a bone marrow biopsy suggested that the red and white blood cell counts were reduced and some blood cells were hemophagocytic. Other tests related to HLH were conducted, and the results were: soluble interleukin-2 receptor alpha chain, 20,229 pg/ml (normal range <6,400 pg/ml); natural killer cell activity, 14.43% (normal range ≥15.11%); IL-10, 1,125.41 pg/ml (normal range <4.91 pg/ml); IL-6, 55.06 pg/ml (normal range <5.30 pg/ml); tumor necrosis factor α (TNF- α), 3.11 pg/ml (normal range <2.31 pg/ml). The IL-2 and IL-4 were within the normal range. In line with the HLH-2004 standard and a hemophagocytic (H) Score cut-off of 190 (**Table 2**), the patient was diagnosed with HLH. The tumor markers of this patient were all negative, including carbohydrate antigen 724, cytokeratin 19 fragment, carcinoembryonic antigen, carbohydrate antigen 242, carbohydrate antigen 125, neuron-specific enolase, alpha-fetoprotein, free human chorionic gonadotropin, squamous cell carcinoma antigen, total prostate-specific antigen, free prostate-specific antigen, and carbohydrate antigen (199). No abnormal space-occupying lesions were found in imageology tests of each site, so tumor-related HLH was excluded first. The results of series of tests, including one for respiratory tract infection pathogens, a tuberculosis infection T cell spot test, a cytomegalovirus PCR assay, and an EBV PCR assay were all negative. Finally, the patient was diagnosed as having HLH associated with *Klebsiella pneumoniae*.

**Table 2:** Diagnosing HLH with A and B, respectively.

A: Diagnostic criteria for HLH according to the conduct of the HLH-2004 trial	
The diagnosis of HLH may be established by:	
a. A molecular diagnosis consistent with HLH: Pathologic mutations of PRF1, UNC13D, Munc18-2, Rab27a, STX11, SH2D1A, or BIRC4	
OR: b. Five out of the eight criteria listed below are fulfilled:	This patient (YES/NO)
Fever 38.5 or more	YES
Splenomegaly	NO
Peripheral blood cells decreased, and matched at least two of the following items: hemoglobin less than 9g/dl (for infants less than 4 weeks, less than 10g/dl); platelet less than 100 ×10^9^/L; the absolute neutrophil count less than 1 × 10^9^/L.	NO
Hypertriglyceridemia (fasting triglyceride > 265mg/dl) and/or hypofibrinogenemia (fibrinogen < 150mg/dl)	NO
Hemophagocytosis in bone marrow or spleen or lymph nodes or liver	YES
Low or absent NK-cell activity	YES
Ferritin >500 ng/ml	YES
Elevated Soluble CD25 (soluble IL-2 receptor alpha): more than 2 standard deviations higher than the age adjusted reference standard of specific laboratory	YES
**B. Score for HLH**	
This scoring system is freely available online (http://saintantoine.aphp.fr/score/).	
Standard for evaluation	The patient’s condition
Known underlying immunodepression	NO
Maximal Temperature (C)	Between 38.4 and 39.4
Hepatomegaly	NO
Splenomegaly	NO
Lower Hemoglobin level	Strictly greater than 9.2g/dl
Lower Leucocytes count	Less than or equal to 5000/mm³
Lower Platelets count	Less than or equal 110000/mm³
Higher Ferritin level (ng/ml)	Between 2000 and 6000
Higher Triglyceride level (mmol/l)	Between 1.5 and 4
Lower Fibrinogen level (g/l)	Strictly greater than 2.5
Higher SGOT/ASAT level (UI/L)	Greater than or equal to 30
Hemophagocytosis features on bone marrow aspirate	YES
**HScore**	190
**Probability of having HS (%)**	80.05922431513314

To identify the focus of the infection and devise an appropriate treatment plan, a whole-hospital discussion was carried out. Heart-related infections were excluded because there is no infective endocarditis or valvular heart disease was found with echocardiography. After comparing the lung CT before and after admission, the lung inflammation was mild and showed little change, so it was not consistent with HLH caused by primary infection. Liver abscess was also excluded by abdominal CT and color Doppler ultrasound. And patient’s acute liver injury was caused by HLH. According to the patient’s routine urine test and urine culture, the acute kidney injury of the patient was associated with multiple organ failure caused by HLH. Finally, the following plan was made: platelets, 1 IU, qd, for one week; dexamethasone, initially 10 mg/m^2^, qd, for two weeks, 5 mg/m^2^, qd, for two weeks, 2.5 mg/m^2^, qd, for two weeks, 1.25 mg/m^2^, qd, for one week; and meropenem, 0.5 g, qd, for two weeks. Due to economic reasons, the patient refused intravenous immunoglobulin.

**Figure 1:**
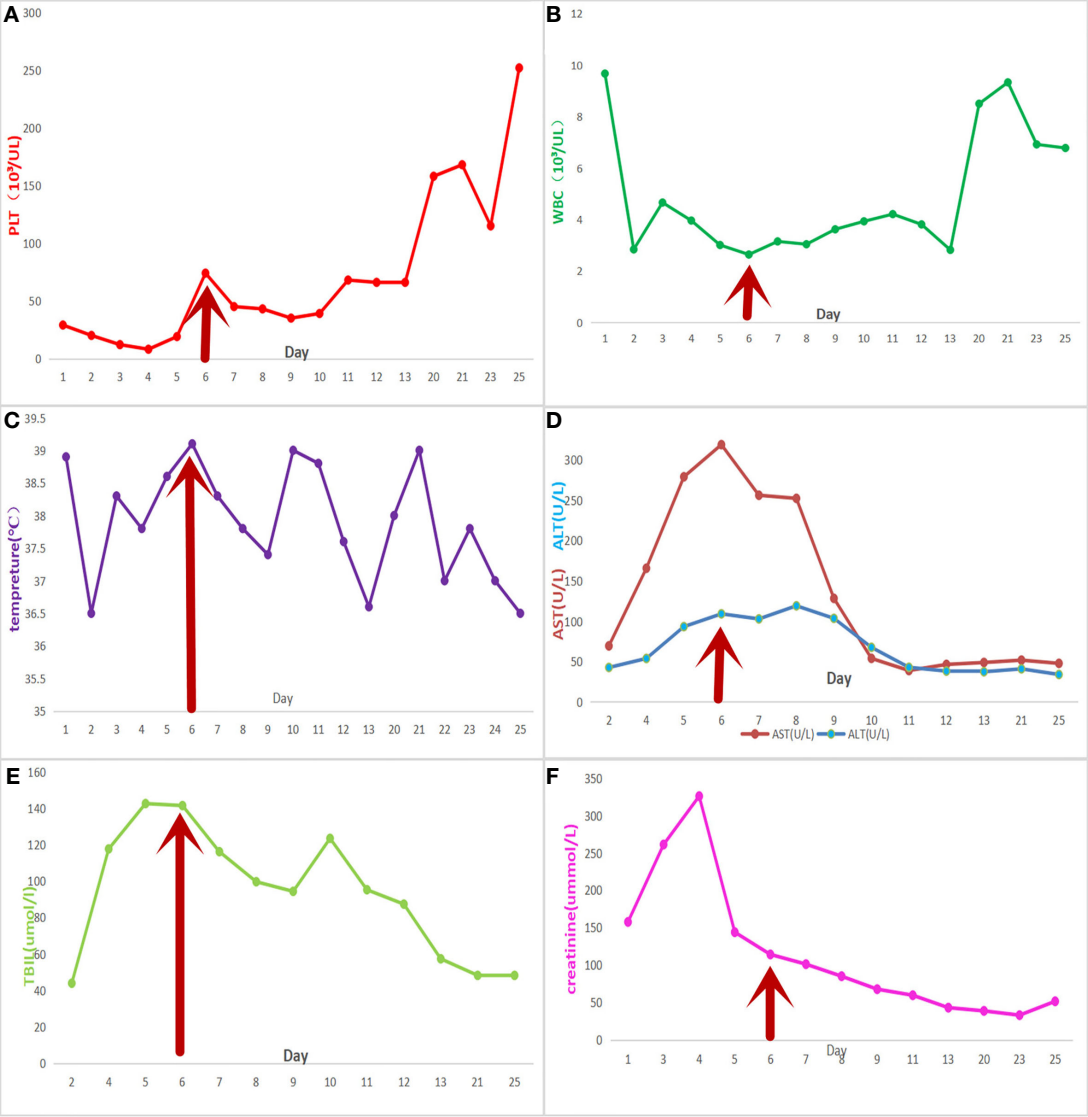
Meropenem was used from the third day onwards. The red arrow represents the start time of the final treatment plan. The patient was discharged on the 13th day and was hospitalized again on the 20th day. **(A)** platelet count; **(B)** white blood cell count; **(C)** daily maximum temperature; **(D)** liver function tests, aspartate transaminase, and alkaline phosphatase; **(E)** total bilirubin level; and **(F)** creatinine levels.

The changes in significant laboratory test results during the whole treatment process are shown in **Figure 1**. By the third day of dexamethasone treatment, the symptoms of general pain had disappeared. The platelets increased gradually in the following week, while ferritin decreased to 1,150.6 μg/L, and serum creatinine, transaminase, and body temperature returned to normal levels.

**Figure 2:**
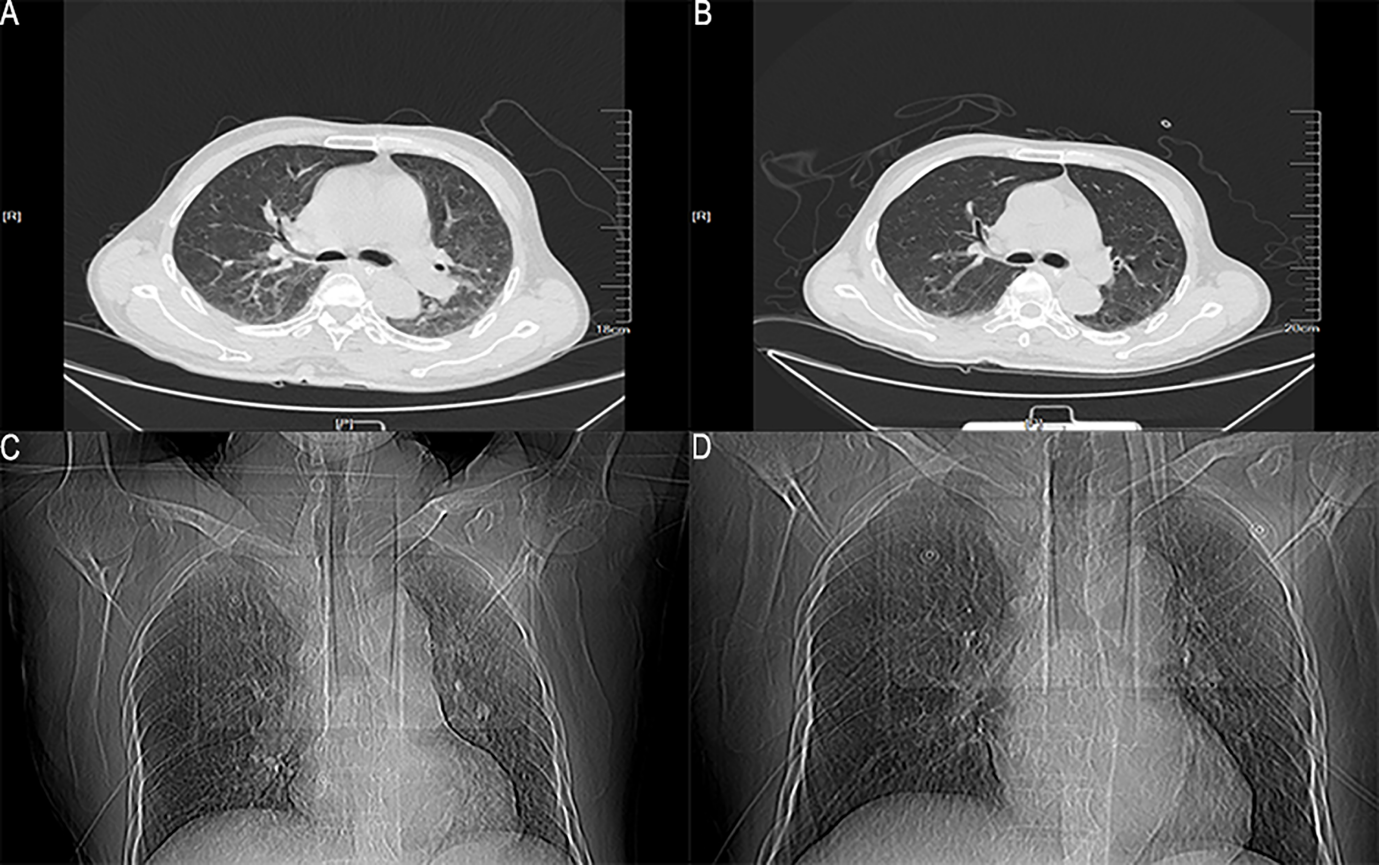
The lung window and location image of the patient’s lung on CT. **(A, C)** The lung CT image on the 6th day after admission; **(B, D)** The lung CT image on the 13th day after admission.

**Figure 3:**
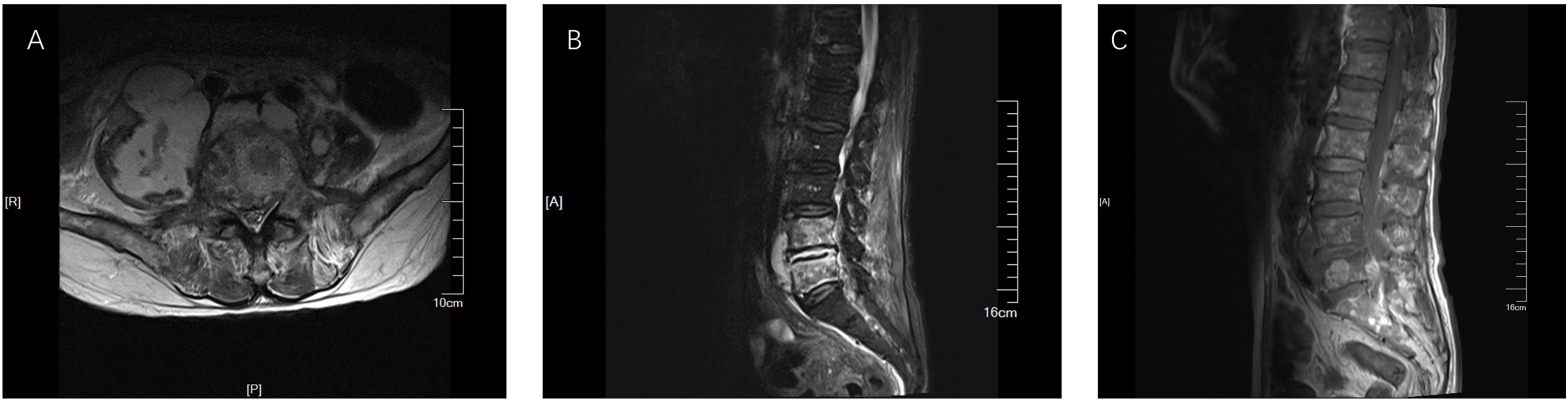
MRI results of lumbar plain scan. **(A)** The result of TIWI; **(B)** The result of T2FS; **(C)** The result of T2WI. The right lumbar vertebrae 4 and 5 showed high signal, and the signal intensity of L4-5 and L5-S1 increased. Abnormal signal was seen around lumbar 4-5 vertebral body, T1WI was equal signal, T2WI was slightly high signal, and lipid pressure image was high signal. The right psoas major, iliopsoas major, and left psoas major were swollen. Irregular abnormal signal shadow was seen in the course area, with uneven internal signal. The lesions on the right side were large, and the maximum axial plane was about 8.1 × 6.0cm.

However, one week later, the patient has fever, lower back pain, and knee pain again. Although laboratory tests and the blood culture results were normal. A repeat CT of the lungs revealed that the inflammation was better than before (**Figure 2**). The patient mainly had lower back pain, and the magnetic resonance imaging (MRI) of the lumbar spine indicated that the possibility of abscess formation in L4 and L5 was high, and the possibility of inflammation and abscess formation in the right greater psoas muscle, iliopsoas muscle, and left greater psoas muscle was high (**Figure 3**). The patient and his family members refused any further treatment and asked for him to be discharged. Three days after discharge, the patient underwent abscess drainage at a local hospital, and his condition gradually improved. Six months after discharge, the patient was followed up by telephone, and the patient said there was no fever, general pain, or other symptoms.

## Discussion and Conclusions

The proportion of bacterial infections in adult HLH is very small, and most of them are related to tuberculosis (4). In this case report, *Klebsiella pneumoniae* is a rare factor for secondary HLH, which has been reported only a few times (5). *Klebsiella pneumoniae* is a common opportunistic pathogen, when the host’s immune capacity decreases it can induce infection and abscesses in many locations. We know from some case reports that *Klebsiella pneumoniae* can cause a psoas abscess and spinal infection, and, in addition, patients with psoas abscesses usually have fever and back or hip pain, but both are very rare (6, 7). Because of the following reasons, the possibility of abscesses caused by other reasons (such as autoantibodies against IL-12 or insufficient haploid function of GATA2) was minimal. First, the patient had been treated with meropenem before the second admission, all indicators improved, and the blood culture was negative when he was re-admitted. Second, it is almost impossible for patients to infect other bacteria outside the hospital. Third, the patient had no discomfort after abscess drainage. Thus, we concluded that it had been caused by the initial spread of *Klebsiella pneumoniae*, resulting in bilateral psoas abscesses and intraspinal abscess, but not relapse or aggravation of HLH. This suggested that, initially, the patient had HLH associated with *Klebsiella pneumoniae*.

The common pathogenesis of primary and secondary HLH is that the body cannot control the excessive activation of macrophages, natural killer (NK cells), and CTLs, resulting in fatal hyperinflammatory syndrome (especially interferon-gamma (IFN-γ), TNF-α, Interleukin (IL)-6, IL-10, IL-18, IL-12, and the activation of soluble IL-2 receptor (SIL-2R) (8–11). Dysfunction or deletion of perforin, caused by congenital genetic defects or acquired triggers, and dysfunction of Fas-dependent dendritic cells can lead to the aggregation of dendritic cells, excessive activation of T cells, excessive production of IFN-γ by CD8 T cells (for most cytotoxic T lymphocytes), and the release of a variety of inflammatory factors (12, 13). During HLH, IFN-γ triggers phagocytosis of macrophages (14). Under normal conditions, NK cells and CTLs scavenge activated macrophages by perforin-dependent cytotoxic action; however, during HLH, NK cells cannot scavenge activated macrophages (15). Then the over-activated macrophages, which cause cytokine production (such as TNF-α, IL-6, IL-10, IL-18, and IL-12), increase significantly (16). The imaging examination of all parts of the patient could not explain his pain symptoms, and the level of IL-6 and IL-10 increased significantly. Therefore, we considered that it was likely induced by a cytokine storm.

Adult HLH is usually difficult to distinguish from sepsis and certain immune-related diseases (such as systemic lupus erythematosus, Still’s a disease, and lymphoma). The diagnosis of sepsis must include positive blood culture etiology and rarely involves multilineage hemocytopenia. However, HLH can be caused by various reasons, and bacterial infection is not a common trigger cause or a necessary standard for the diagnosis of HLH.

In terms of a single criterion, compared with the other seven diagnostic HLH-2004 criteria, thus, ferritin appears to be a more reliable parameter for screening for HLH (17). We also found that elevated ferritin was a common manifestation of adult HLH (18). The numerical value of elevated ferritin in HLH patients was often much higher than in patients with infection, inflammation, or liver disease (19). Therefore, ferritin can play an essential role in the overall diagnosis of HLH (20). In this case, early multi-disciplinary consultation, the ferritin test, and our special vigilance concerning the elevated ferritin result helped us make a definitive diagnosis quickly. Therefore, we now believe that it is necessary to include ferritin in the monitoring of the ICU admission team of every department to reduce the rate of missed diagnosis.

The treatment of HLH is based on the principle of eliminating trigger factors and controlling excessive immune response (21). For immune control, an HLH-94 plan is currently recognized as the HLH-specific therapy, and it can significantly improve the prognosis of HLH. In this plan, chemoimmunotherapy is used to inhibit the life-threatening inflammatory response (22). However, not all patients should receive HLH specific treatment. In the case of infection-induced HLH, the formulation of the treatment plan depends on many factors. First, the formulation of the plan depends on different triggers. For example, HLH-94 protocol plus Rituximab have been proven effective for EBV-HLH, but, for intracellular infectious diseases, specific antimicrobial agents are needed rather than HLH specific treatment similar to HLH-94. Second, advanced age, thrombocytopenia, prolonged activated partial thromboplastin time, hypertriglyceridemia, elevated lactate dehydrogenase, and malignant tumors are risk factors for the early death of HLH adults, so the early death risk assessment of adult HLH patients plays a guiding role in the treatment intensity and timing (23). There is no doubt about the effect of antibacterial treatment for bacteria-induced HLH, but there are few clinical reports about it as it is very rare. Therefore, there is no specific standard about whether to carry out HLH specific treatment and how to carry it out. Some cases of *Mycoplasma pneumoniae*-induced HLH have demonstrated that, for patients in a stable condition or mild to moderate infection, a complete HLH-94 plan was not needed, and corticosteroids or cyclophosphamide alone, or one of them combined with antibiotic treatment was also effective (24). Considering the patient’s advanced age and the side effects of cytotoxic drugs, although the patient’s condition was not stable, we still decided to use meropenem combined with dexamethasone first. We then chose whether or not to use the HLH-94 plan depending on whether there was a change in the patient’s condition. The final result proved that this incomplete HLH specific therapy combined with antibiotics was effective for this patient.

Another excellent treatment for HLH in infected patients is intravenous immunoglobulin (25). In addition, the efficacy of some targeted drugs has been confirmed, such as INF-γ inhibitors (Emapalumab), Interleukin-6 inhibitors (tocilizumab), and Janus kinase/activator of signal transduction and transcription (JAK-STAT) inhibitors (Ruxolitinib), which provide more choices for the treatment for HLH (26, 27). However, it is worth noting that all the above-mentioned treatments have their scope of application, and doctors need to choose a plan according to the condition of patients. There is a lot of room to explore in the treatment of HLH, and I believe there will be more discoveries in the future.

## Data Availability Statement

The original contributions presented in the study are included in the article/supplementary material. Further inquiries can be directed to the corresponding author.

## Ethics Statement

The studies involving human participants were reviewed and approved by The First Hospital of Jilin University ethics committee. The patients/participants provided their written informed consent to participate in this study. Written informed consent was obtained from the individual(s) for the publication of any potentially identifiable images or data included in this article.

## Author Contributions

YZ conceived the idea and conceptualized the study. JL collected the data. JW analyzed the data. YZ drafted the manuscript, then YW reviewed the manuscript. All authors contributed to the article and approved the submitted version.

## Conflict of Interest

The authors declare that the research was conducted in the absence of any commercial or financial relationships that could be construed as a potential conflict of interest.
